# Composition and Effects of Aerosol Particles Deposited on Urban Plant Leaves in Terrestrial and Aquatic Habitats

**DOI:** 10.3390/plants13213056

**Published:** 2024-10-31

**Authors:** Siqi Chen, Fangmin Fei, Yaobin Song, Ming Dong, Aiping Wu, Hua Yu

**Affiliations:** 1 Key Laboratory of Hangzhou City for Ecosystem Protection and Restoration, College of Life and Environmental Sciences, Hangzhou Normal University, Hangzhou 311121, China; chensq0412@163.com (S.C.); feifangmin@163.com (F.F.); ybsong@hznu.edu.cn (Y.S.); dongming@hznu.edu.cn (M.D.); 2 Hunan Provincial Key Laboratory of Rural Ecosystem Health in Dongting Lake Area, Ecology Department, College of Environment and Ecology, Hunan Agricultural University, Changsha 410128, China; wuaip8101@126.com

**Keywords:** aerosol particles, amphibious plants, leaf functional traits, particle deposition, terrestrial and aquatic habitats

## Abstract

Plants play a vital role in mitigating aerosol particles and improving air quality. This study investigated the composition characteristics and potential effects of particles retained on the leaf surfaces of two amphibious plants (i.e., *Alternanthera philoxeroides* and *Hydrocotyle vulgaris*) in both terrestrial and aquatic habitats. The results show that plant habitats influenced the composition of aerosol particles retained on leaf surfaces. Specifically, plants in terrestrial habitats retained a higher mass concentration of coarse and large particles rich in inorganic Ca^2+^, accounting for over 70% of total ions, whereas plants in aquatic habitats retained a greater abundance of fine and secondary particles with high fractions of water-soluble NO_3_^−^ and SO_4_^2−^, taking up over 65% of total anions. Secondary particles deposited on the surfaces of plants in aquatic habitats tend to deliquesce and transform from the particle phase to the liquid phase. Terrestrial habitats facilitate the deposition of large particles. Additionally, particle accumulation on leaf surfaces adversely affected the stomatal conductance of plant leaves, leading to reductions in both the transpiration and photosynthetic rates. This study provides insights into the impact and role of plants from different habitats in mitigating urban particulate pollution.

## 1. Introduction

Atmospheric particulate pollution has emerged as a significant environmental concern due to its potential health and environmental hazards [1,2], drawing widespread global attention. Aerosol particles comprise viruses, bacteria, heavy metals, polycyclic aromatic hydrocarbons (PAHs), and secondary aerosol particles [3,4,5], and prolonged exposure to particulate pollution can result in various health issues, including respiratory and cardiovascular diseases, lung cancer, and coronary heart disease [6,7]. For instance, PM_2.5_ pollution led to 4.14 million deaths worldwide in 2019, constituting 62% of all air pollution-related deaths [8]. Therefore, it is imperative to identify effective solutions to ameliorate urban atmospheric pollution and safeguard public health.

Many studies have highlighted the important role that urban plants play in alleviating particulate pollution [9,10,11,12]. Plant leaves have great potential to capture and retain aerosol particles, thereby reducing the mass concentration of particulate pollution in the atmosphere [13]. Previous research has estimated that urban vegetation can remove 8–8.4 kg ha^−1^ yr^−1^ of PM_10_ in the urban parks of Italy [10], 7.5 kg ha^−1^ yr^−1^ of PM_2.5_ for *Pinus sylvestris* in Belgium [14], and 11.9 *t* km^−2^ yr^−1^ of PM_2.5_ for *Cedrus deodara* in China [15]. Additionally, Pettit et al. [16] reported that green vegetation along urban roadsides can reduce the PM_2.5_ concentration by 22.5% in ambient air. Therefore, green plants are effective in mitigating particle pollution in urban air.

The particle retention efficiency of plants is directly influenced by leaf traits [17,18]. Previous studies have found that leaf functional traits and morphological characteristics, such as the trichome, wax, stoma, and groove of the leaf surface, can impact the particle retention efficiency of plants [19,20,21,22]. Chen et al. [13] reported that plant leaves with a larger stomatal area, lower stomatal density, smaller specific leaf area, and higher epicuticular wax content are more effective at retaining aerosol particles. Additionally, coniferous trees have a higher particle retention efficiency compared to broadleaved trees [23]. Significant differences exist in the particle retention capacity among plants, including trees, shrubs, and herbs [24,25].

While numerous studies have demonstrated that plants can effectively alleviate particulate pollution [26,27], the impacts of aerosol particles on plants have rarely been thoroughly clarified. Upon deposition on leaves and entry into stomata, aerosol particles tend to exert adverse effects on plant physiology, with stomata being particularly vulnerable and sensitive to particle deposition [28]. Particles accumulate on the foliar surface of plants through dry or wet deposition, which affects the stomatal conductance and decreases the photosynthetic rate [29,30]. The long-term retention of particles on plant leaves leads to the degradation of epicuticular wax, induces changes in leaf wettability, and inhibits plant transpiration [28,31]. Thus, it is necessary to systematically study the responses of plants to particle accumulation.

Currently, studies primarily focus on the particle retention efficiencies of trees and shrubs in urban terrestrial habitats [26,32]. Aquatic habitats differ from terrestrial habitats in environmental factors, such as high humidity and rapid evaporation, which influence the deposition of aerosol particles on plant leaves [17,33,34]. However, despite being an important component of urban vegetation, plants in aquatic habitats have rarely been investigated in terms of particle deposition. In this study, two plant species, *Alternanthera philoxeroides* and *Hydrocotyle vulgaris*, which are amphiphytes in terrestrial and aquatic habitats, were selected as the studied species. Understanding the interactions between particle accumulation and plant functional traits in different habitats is essential for the selection and configuration of urban greenery plants. The aims were to test the following hypotheses: (1) the composition characteristics of deposited particles vary in different habitats; (2) habitats affect the particle retention efficiencies of plants; (3) the potential impacts of accumulated particles vary across plants to habitats.

## 2. Materials and Methods

### 2.1. Studied Species

*Alternanthera philoxeroides*, commonly known as alligator weed, is a perennial amphibious plant in the family Amaranthaceae, native to the temperate regions of South America [35]. Its leaves are characterized by a glabrous or appressed hair surface on the adaxial side, while granular projections are present on the abaxial side. It is widely distributed in both terrestrial and aquatic habitats.

*Hydrocotyle vulgaris* is a perennial amphibious plant in the family Umbelliferae, native to semi-moist to wet habitats across Europe and parts of Northwest Africa [36]. Its leaves are glabrous on both surfaces, covered with a shiny wax. This species is widespread in both terrestrial and aquatic habitats in China [37].

### 2.2. Sampling Sites

The sampling sites for this study are located in the Cangqian Campus of Hangzhou Normal University (30°17′24″ N, 120°0′29″ E), in Hangzhou City of Zhejiang Province, Eastern China. This area includes terrestrial and aquatic habitats with a similar atmospheric environment, and represents a typical urban environment surrounded by main roads without any high-polluting industry nearby. The primary sources of air pollutants are attributed to dense traffic. A river runs through the campus, with the sampling sites for the aquatic habitats positioned along the river’s edge. In contrast, the sampling sites for the terrestrial habitats are situated in a grassy area approximately 100 m away from the river, with a similar atmospheric environment.

### 2.3. Sample Collections

Samples were collected between 9:00 and 11:00 a.m. on 31 December 2021, with no rainfall observed for a week preceding the collections of leaf samples. Five individual plants in good growth conditions and free of pests and disease were randomly selected from each species (*A. philoxeroides* or *H. vulgaris*) and each habitat (terrestrial or aquatic), with four leaves collected per individual plant at a height of 20 cm. Twenty samples per treatment were placed in labeled Petri dishes and immediately transported to the laboratory. Subsequently, the samples were dried at 60 °C for 24 h until reaching a constant weight for subsequent measurements.

### 2.4. Measurements of Particle Compositions

The collected particles were categorized based on their sizes into fine particles (*Φ* ≤ 2.5 μm), coarse particles (2.5 < *Φ* ≤ 10 μm), and large particles (*Φ* > 10 μm), as well as the total suspended particulate (TSP), representing the sum of all particles deposited on leaf surfaces. The leaf samples were dried in an oven at 60 °C until reaching a constant weight, in preparation for the observations of the particle types and elemental compositions using scanning electron microscopy coupled with energy-dispersive X-ray spectroscopy (SEM-EDS).

Fragments of 1 cm × 1 cm in size were randomly cut from ten leaf samples per plant species, and then they were divided into two halves, for the observation of the adaxial and abaxial surfaces of the plant leaves, respectively. The surfaces of the fragments were coated with gold using an ion sputter coater (KYKY SCB-12, Beijing, China), following the procedure described by our previous research [13], before being observed using SEM-EDS (Phenom XL, Phenom-World, Eindhoven, The Netherlands) in low-vacuum mode at 15 kV. Five spots were randomly selected on each leaf fragment of the leaf samples, and their images were acquired at magnifications ranging from 2000× to 20,000× with a high resolution.

(1) Number density of particles

Using SEM images, the ImageJ/FIJI V2.1.0 (National Institutes of Health, Bethesda, MD, USA) (https://imagej.net/software/fiji/, accessed on 18 August 2024) was employed to quantify the number density and size distribution of the particles deposited on both the adaxial and abaxial surfaces of the leaf samples for the plant species in the terrestrial and aquatic habitats. The number density of the particles, including fine particles, coarse particles, large particles, and the TSP, referred to as the particle density (10^5^ cm^−2^), was calculated as follows [38]:

(1) $$\mathrm{Particle}\;\mathrm{density}=\frac{\mathrm{Particle}\;\mathrm{number}}{\mathrm{SEM}\;\mathrm{image}\;\mathrm{area}}$$

(2) Mass concentration of particles

The particle mass per unit leaf area, referred to as the mass concentration (μg cm^−2^) of the particles (including fine particles, coarse particles, large particles, and the TSP), was calculated using the following equation [39]:

(2) $$V_{i}=\frac{4}{3}\pi (\frac{D}{2}{)}^{3}$$

(3) $$\mathrm{Particle}\;\mathrm{mass}=\frac{\sum_{i}V_{i}\text{·}\rho }{\mathrm{SEM}\;\mathrm{image}\;\mathrm{area}}$$

where

*V_i_* is the particle volume;

*D* is the particle diameter;

*ρ* is the average density of the particles, with the value here being 2 g cm^−3^.

(3) Concentration of water-soluble inorganic ions

For the analyses of water-soluble inorganic ions, ten leaves of each species per treatment were selected. To extract water-soluble ions from the deposited particles on the leaves, each leaf sample was placed in a centrifuge tube containing 50 mL of deionized water, and then shaken at 30 rpm for 1 h in a shaker. The resulting solution was filtered by using a 10 mL syringe equipped with a 2.2-μm strainer. An ion chromatograph (Dionex ICS-600; Thermo Fisher Scientific, Waltham, MA, USA) was utilized to determine the concentrations of major water-soluble inorganic ions, including Ca^2+^, K^+^, NH_4_^+^, Na^+^, SO_4_^2−^, NO_3_^−^, Cl^−^, and F^−^. To exclude the impacts of the plant leaves on the concentration measurement of water-soluble inorganic ions in the deposited particles, we selected a leaf that removed deposited particles as a blank. A soft brush was used to gently remove the particles from the leaves immediately after sample collection, ensuring that the blades remained undamaged.

### 2.5. Measurements of Leaf Functional Traits

(1) Photosynthetic-related parameters

Prior to each sample collection, the LICOR 6400 Photosynthetic System (Li-6400) was utilized to measure the photosynthetic activity of the plants, including the leaf temperature (*T_f_*), photosynthetic rate (*P_n_*), stomatal conductance (*G_s_*), and transpiration rate (*T_r_*). The chlorophyll content was measured using a chlorophyll meter, the SPAD 502 plus (Konica Minolta, Japan). For these measurements, 10 leaves per treatment (i.e., plants and habitats) were randomly selected, with each leaf measured in triplicate.

(2) Morphological characteristics

Based on the images of the leaf samples obtained using the EPSON V700 scanner (Seiko Epson, Nagano, Japan), the leaf area (LA, cm^2^) was measured with the WinFOLIA software (Pro 2013a; Regent Instruments Inc., Quebec, QC, Canada), with ten replications. The specific leaf area (SLA, cm^2^ mg^−1^) was calculated as described in a previous study [13].

After drying the leaf samples at 60 °C for 24 h until they reached a constant weight, they were weighed and recorded as *m*_1_ (mg). The dried leaf samples were completely soaked in trichloromethane for two minutes, and then they were placed in the oven until the liquid had evaporated completely. Finally, they were reweighted and recorded as *m*_2_ (mg). The wax content (WC, mg cm^−2^) was calculated according to the following equation:

(4) $$WC=\frac{m_{1}-m_{2}}{LA}$$

where

*m*_1_ is the dry biomass of a leaf sample;

*m*_2_ is the dry biomass after wax removal;

*LA* is the leaf area of the leaf sample.

### 2.6. Data Analysis

The data analysis and graphics were performed using SPSS 26.0 statistical software (SPSS Inc., Chicago, IL, USA) and Origin 2023 software (Origin Lab Corp., Northampton, MA, USA). Before conducting the analyses, the normal distribution was verified using the Shapiro–Wilk test. A two-way analysis of variance (two-way ANOVA) and Duncan’s multiple comparison test were conducted to detect the differences in particle characteristics and plant leaf traits. Principal component analysis (PCA) was adopted to demonstrate the effects of the mass concentrations and ion compositions of the deposited particles. Spearman’s correlation analysis was employed to illustrate the correlations between the particle mass and photosynthetic-related parameters.

## 3. Results

### 3.1. The Size Distributions of Deposited Particles

**Figure 1:**
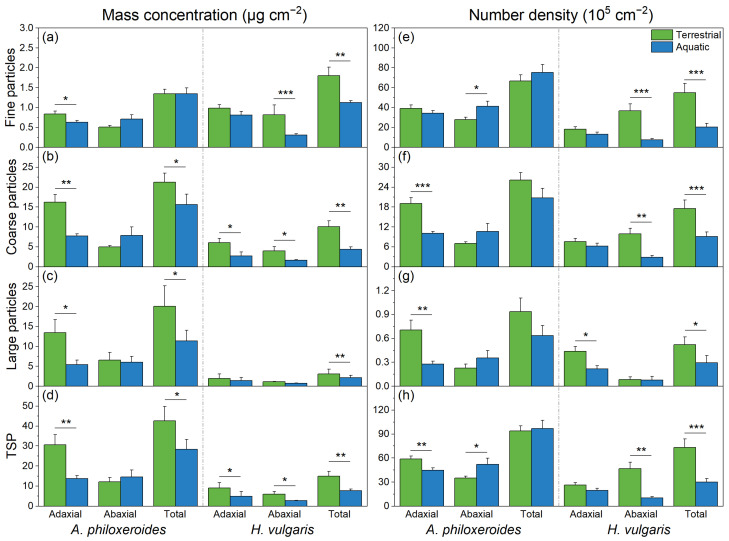
The mass concentrations and number densities of fine particles (**a**,**e**), coarse particles (**b**,**f**), large particles (**c**,**g**), and the TSP (**d**,**h**) retained on the adaxial and abaxial leaves of *Alternanthera philoxeroides* and *Hydrocotyle vulgaris* in the terrestrial habitats (green bar) and aquatic habitats (blue bar), respectively. The data are presented as the Mean ± SE. *—*p* < 0.05; **—*p* < 0.01; ***—*p* < 0.001.

The distributions of the deposited particles varied with the plant species and habitat treatments, affecting both the mass concentrations and number densities (Table 1; Figure 1). Specifically, the different habitats significantly affected the mass concentration and number density of the fine particles and TSP, while the plant species significantly influenced the mass concentration and number density of the fine particles, coarse particles, large particles, and TSP. Moreover, the interactions between the plant species and habitat treatments significantly impacted the mass concentration of fine particles and the number density of coarse particles, as well as the mass concentration and number density of large particles and the TSP (Table 1).

In comparison, more particles were deposited on the leaves of the plants in the terrestrial habitats than in the aquatic habitats (Figure 1b–d,f,g), although there was no significant difference in the fine particles on the leaves of *A. philoxeroides* between the different habitats (Figure 1a,e). Additionally, more particles (e.g., fine particles, coarse particles, large particles, and the TSP) tended to retain on the adaxial surface than on the abaxial surface of the plant leaves in the terrestrial habitats (Figure 1), and the mass concentrations of coarse particles, large particles, and the TSP on *A. philoxeroides* were higher than those on *H. vulgaris* (Figure 1a–d). However, the mass concentrations and number densities of particles on the abaxial surface of *A. philoxeroides* in the aquatic habitats were slightly higher than in the terrestrial habitats.

**Figure 2:**
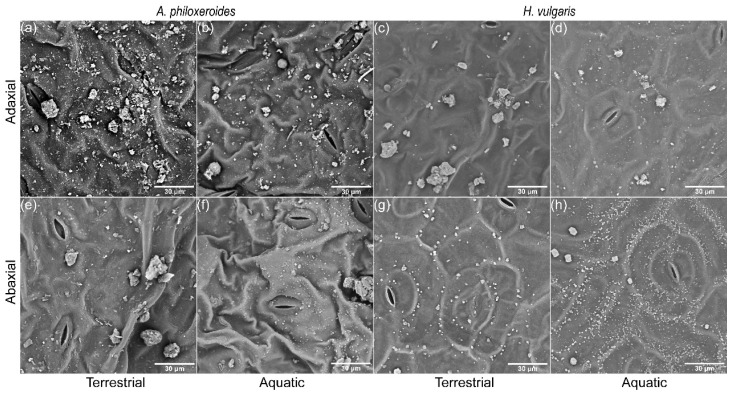
Scanning electron microscopy (SEM) images of the retained particles and the micro-morphological characteristics for the adaxial (**a**–**d**) and abaxial (**e**–**h**) leaf surfaces of *A. philoxeroides* (**a**,**b**,**e**,**f**) and *H. vulgaris* (**c**,**d**,**g**,**h**) in terrestrial habitats (**a**,**c**,**e**,**g**) and aquatic habitats (**b**,**d**,**f**,**h**) at ×2000 magnification, respectively.

Based on the high-resolution images from the SEM-EDS, numerous aerosol particles of various sizes and shapes were distributed on the leaf surfaces, varying with the plant species and habitat treatments (Figure 2). For both *A. philoxeroides* and *H. vulgaris*, more coarse and large particles were present on the adaxial surface of the plant leaves (Figure 2a–d), while the abaxial surface had numerous fine particles. The deposited particles on the leaf surfaces in the terrestrial habitats were dominated by large and coarse particles (Figure 2a,c,e,g). However, the particles in the aquatic habitats were mainly fine particles (Figure 2b,d,f,h), appearing as smaller and more regular than those in the terrestrial habitats, especially on the abaxial surface of the plant leaves in the aquatic habitats (Figure 2f,h).

### 3.2. The Composition Characteristics of Deposited Particles

**Figure 3:**
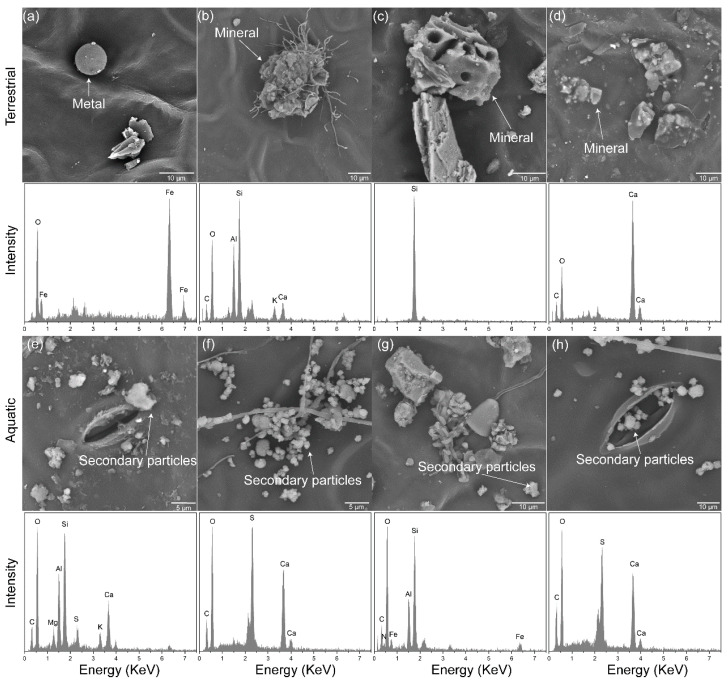
High-resolution images obtained by SEM-EDS showing the main types of retained particles on the leaf surfaces. These include a spherical metal (Fe) particle (**a**), mineral particles (**b**–**d**), as well as secondary particles (**e**–**h**) aggregating on the leaf surfaces or existing inside and outside of the stomata in the terrestrial habitats (**a**–**d**) and aquatic habitats (**e**–**h**), respectively, at high magnifications of ×6000 and ×10,000.

The composition characteristics of the retained particles on the leaf surfaces exhibited significant differences between the terrestrial and aquatic habitats (Figure 3). For example, in the terrestrial habitats, the particles on the plant leaves were mainly composed of mineral particles and metal particles, containing elements such as Ca, Si, Fe, O, C, and K (Figure 3a–d). In contrast, the aquatic habitats had an abundance of secondary particles, with elements primarily including Ca, Si, S, O, and C (Figure 3e–h). Additionally, most of the particles on the plant leaves in the terrestrial habitats existed as single and separated particles, whereas those in the aquatic habitats mostly formed dense clusters.

**Figure 4:**
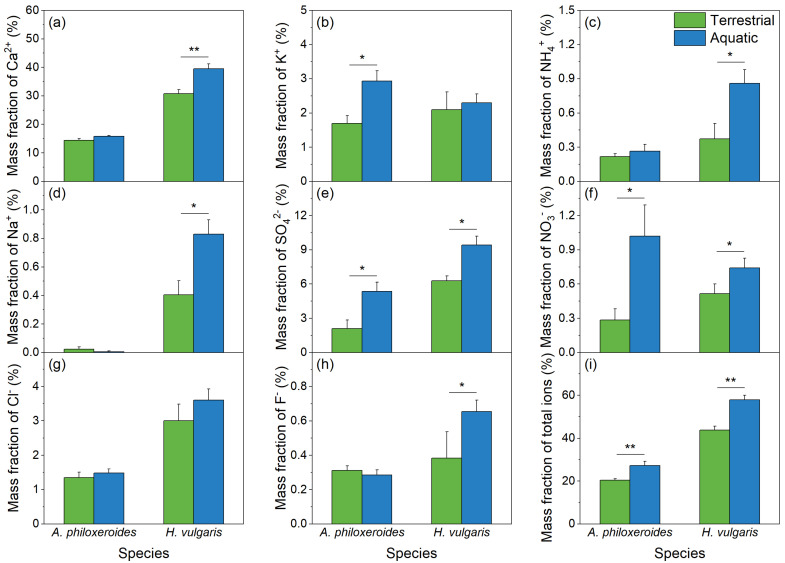
Mass fractions of water-soluble inorganic ions, including Ca^2+^ (**a**), K^+^ (**b**), NH_4_^+^ (**c**), Na^+^ (**d**), SO_4_^2−^ (**e**), NO_3_^−^ (**f**), Cl^−^ (**g**), and F^−^ (**h**), and the total ions (**i**), in the particles retained on the leaf surfaces of *A. philoxeroides* and *H. vulgaris* in the terrestrial habitats (green bar) and aquatic habitats (blue bar), respectively. The data are presented as the Mean ± SE. *—*p* < 0.05; **—*p* < 0.01.

The water-soluble inorganic ions in the deposited particles differed between plant species and habitat treatments (Figure 4). Habitat treatments significantly impacted the mass fractions of water-soluble inorganic ions, such as SO_4_^2−^ and NO_3_^−^, while the combination of plant species and habitat treatments significantly affected the mass fraction of F^−^ (Table 1). Interestingly, the mass fractions of water-soluble inorganic ions in the deposited particles from the aquatic habitats were higher than those from the terrestrial habitats, particularly for dominated ions like Ca^2+^ and SO_4_^2−^, as well as the total ions (Figure 4). Additionally, in both the terrestrial and aquatic habitats, the mass fractions of water-soluble inorganic ions in the deposited particles on *H. vulgaris* tended to be higher than those on *A. philoxeroides*, with the exceptions of the mass fractions of K^+^ and NO_3_^−^ in the aquatic habitats (Figure 4b,f).

**Figure 5:**
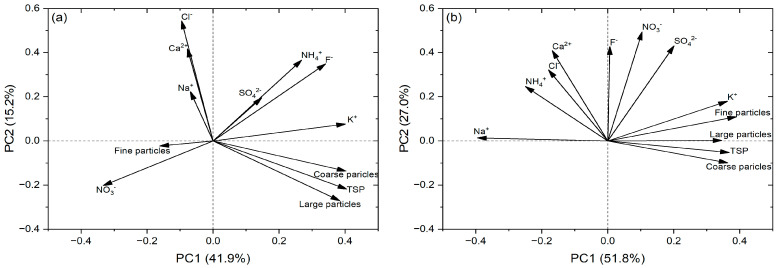
Principal component analysis (PCA) of the mass concentrations of particles with different sizes (i.e., fine particles, coarse particles, large particles, and the TSP) and the mass fractions of water-soluble ions (i.e., Ca^2+^, K^+^, NH_4_^+^, Na^+^, SO_4_^2−^, NO_3_^−^, Cl^−^, and F^−^) retained on the leaf surfaces of the plants in the terrestrial habitats (**a**) and aquatic habitats (**b**), respectively. PC indicates a principal component.

The PCA demonstrated the differences between the terrestrial and aquatic habitats (Figure 5). In the terrestrial habitats, two principal components (PCs) were extracted, with a cumulative contribution of 57.1% (Figure 5a). PC1 accounted for 41.9% of the total variability and was positively correlated with coarse particles, the TSP, large particles, and K^+^, while negatively correlated with NO_3_^−^. PC2 explained 15.2% of the variation and was positively correlated with Cl^−^ and Ca^2+^. In the aquatic habitats, PC1 and PC2 cumulatively contributed 78.8% (Figure 5b). PC1 accounted for 51.8% of the total variability and was positively correlated with fine particles, the TSP, coarse particles, and K^+^, but negatively correlated with Na^+^. PC2 explained 27.0% of the variation and was positively correlated with NO_3_^−^ and SO_4_^2−^. The effects of the mass concentrations and ion compositions of the deposited particles varied between the different habitats.

### 3.3. The Leaf Functional Traits of Plants

**Figure 6:**
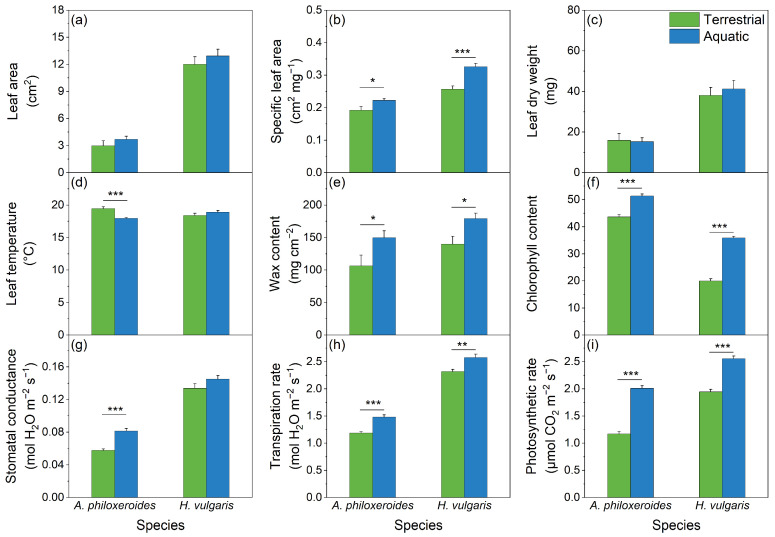
Leaf functional traits, including the leaf area (**a**), specific leaf area (**b**), leaf biomass (**c**), leaf temperature (**d**), wax content (**e**), chlorophyll content (**f**), stomatal conductance (**g**), transpiration rate (**h**), and photosynthetic rate (**i**) of *A. philoxeroides* and *H. vulgaris* in the terrestrial habitats (green bar) and aquatic habitats (blue bar), respectively. The data are presented as the Mean ± SE. *—*p* < 0.05; **—*p* < 0.01; ***—*p* < 0.001.

**Figure 7:**
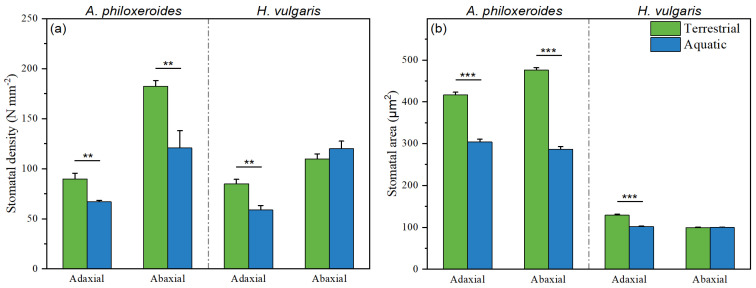
Stomatal density (**a**) and stomatal area (**b**) of *A. philoxeroides* and *H. vulgaris* in the terrestrial habitats (green bar) and aquatic habitats (blue bar), respectively. The data are presented as the Mean ± SE. **—*p* < 0.01; ***—*p* < 0.001.

The leaf functional traits of the plants varied by species and were significantly affected by the habitat treatments (Table 2; Figure 6). Specifically, the leaf area and leaf biomass primarily varied by plant species, while the wax content, stomatal conductance, and transpiration rate were significantly influenced by both the plant species and habitat treatments. Additionally, the specific leaf area, stomatal density, stomatal area, chlorophyll content, and photosynthetic rate were significantly affected by both the plant species and habitat treatments, as well as their interactions (Table 2; Figure 7). Notably, the chlorophyll content of *A. philoxeroides* was significantly higher than that of *H. vulgaris*, and the leaf temperature of both plants was similar. However, other leaf traits of *A. philoxeroides* had lower values than those of *H. vulgaris* in both the aquatic and terrestrial habitats (Figure 6). Moreover, the values of the specific leaf area, wax content, chlorophyll content, transpiration rate, and photosynthetic rate of the plant leaves in the aquatic habitats were significantly higher than those in the terrestrial habitats (Table 3).

### 3.4. Relation Between Particle Deposition and Leaf Functional Traits

**Figure 8:**
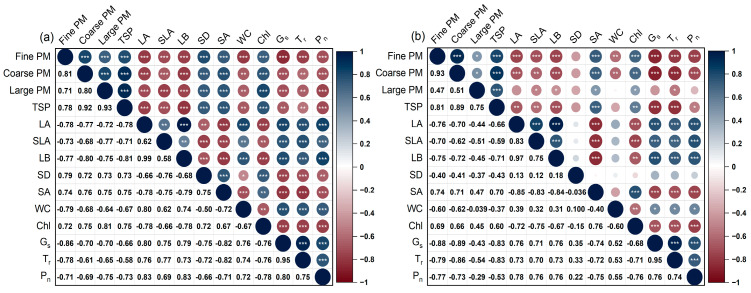
Correlation analysis of the mass concentrations of particles with different sizes (i.e., fine particles, coarse particles, large particles, and the TSP) and leaf functional traits (e.g., leaf area (LA), specific leaf area (SLA), leaf biomass (LB), stomatal density (SD), stomatal area (SA), wax content (WC), chlorophyll content (*Chl*), stomatal conductance (*G_s_*), transpiration rate (*T_r_*), and photosynthetic rate (*P_n_*)) of *A. philoxeroides* and *H. vulgaris* in the terrestrial habitats (**a**) and aquatic habitats (**b**), respectively. *—*p* < 0.05; **—*p* < 0.01; ***—*p* < 0.001.

In this study, the sampling sites were close to each other, ensuring a consistent atmospheric environment and similar sedimentation rates of particulate matter. Therefore, the differences in the retained particles on the leaves mainly comes from the plant habitat. The correlations between the particle mass and leaf functional traits tended to be similar across habitats (Figure 8). Specifically, the deposited particles, including fine, coarse, and large particles, as well as the TSP, were positively correlated with the accumulation of particles of different diameters. Increases in the particle deposition were likely associated with increased stomatal density, stomatal area, and chlorophyll content, while simultaneously reducing the leaf area, leaf biomass, specific leaf area, wax content, stomatal conductance, transpiration rate, and photosynthetic rate. Morphological characteristics, such as the leaf biomass, leaf area, and specific leaf area, were negatively related to the stomatal density, stomatal area, and chlorophyll content, but positively correlated with the wax content, stomatal conductance, transpiration rate, and photosynthetic rate.

## 4. Discussion

### 4.1. Differences in Particle Characteristics Deposited on Plants in Terrestrial and Aquatic Habitats

Particle deposition on plants has been confirmed to be affected by the plant species and leaf texture [40,41,42]. Different habitats exhibit both direct and indirect impacts on the particle deposition on plant species, although there are few studies focusing on this [43]. This study found that the mass concentration and number density of particles deposited on the plants in the terrestrial habitats were higher than those in the aquatic habitats, which was consistent for both *A. philoxeroides* and *H. vulgaris* (Figure 1). Specifically, in the terrestrial habitats, most of the deposited particles were coarse particles and large particles, characterized by primary particles with irregular shapes, such as mineral particles. In contrast, in the aquatic habitats, fine particles were the main type retained on the plant leaves, and most were secondary particles with regular shapes (Table 3). There were significant differences in the size distribution and particle types of the retained particles between the terrestrial and aquatic habitats.

Additionally, compared with the particles deposited in the terrestrial habitats, those from the aquatic habitats had higher mass fractions of water-soluble inorganic ions. This may be due to the high relative humidity (RH) in aquatic habitats, as water-soluble inorganic ions become more abundant with increasing RH [44]. In the terrestrial habitats, the main water-soluble inorganic ion was Ca^2+^ in coarse particles and large particles, accounting for over 70% of total ions, which generally derives from soil, ground dust, and construction dust [24]. In contrast, in the aquatic habitats, water-soluble NO_3_^−^ and SO_4_^2−^ in retained particles, taking up over 65% of total anions, especially fine particles, played a significant role. These ions primarily originate from industrial sources, traffic emissions, fossil fuel combustion, and the secondary transformation of gaseous pollutants in the atmosphere [45]. Additionally, some sulfate-containing components may be derived from nanoparticles released by the plants themselves [46]. The high mass fractions of NO_3_^−^ and SO_4_^2−^ were likely linked to the secondary transformation of gaseous pollutants in the atmosphere [47,48,49,50], indicating that the sources of the deposited particles on the plant leaves in the aquatic habitats come not only from ground dust but also from exhaust emissions and secondary particles.

The differences in the deposited particles between the terrestrial and aquatic habitats can be attributed to water evaporation and the RH. The sample sites in both habitats had very similar environmental factors, such as temperature, sunshine, and atmospheric compositions, which can be excluded as potential sources for the differences in particle deposition. In contrast, the aquatic environment had higher water evaporation and higher transpiration rates of plant leaves, leading to a higher RH and more turbulent airflow. In conditions of higher RH, aerosol particles undergo hygroscopic growth and transform from the particle phase to the liquid phase, making it easier for these particles to be deposited on plant leaves after hygroscopic growth [51,52,53,54]. This process facilitates the accumulation of fine particles on plant leaves in aquatic habitats. Ryu et al. [43] indicated that the increase in RH caused by plant leaf transpiration and environmental water evaporation could enhance the removal of aerosol particles. Moreover, high RH in aquatic habitats promotes heterogeneous reactions of NO_x_ and SO_2_, leading to the transformation of sulfates and nitrates [40,41,42,47]. This could explain the high concentrations of NO_3_^−^ and SO_4_^2−^ in the particles deposited on plant leaves in aquatic habitats.

### 4.2. Effects on Particle Retention Efficiency of Plants by Different Habitats

The particle retention efficiency of plants is closely related to leaf traits, which vary not only by plant species but also by different habitats [55,56]. In this study, based on the leaf traits of *A. philoxeroides* and *H. vulgaris*, plants in aquatic habitats grew significantly better than those in terrestrial habitats (Figure 6). Specifically, the values of the specific leaf area, wax content, chlorophyll content, transpiration rate, and photosynthetic rate of the plant leaves in the aquatic habitats were significantly higher than those in the terrestrial habitats. Moreover, this study discovered significant negative correlations between the mass of deposited particles and leaf traits, such as the leaf area, specific leaf area, and wax content. These factors are important in causing inter-species differences in particle capture and retention, with higher particle accumulation on smaller and thicker leaves covered with less wax. This is consistent with the findings that a single leaf with a smaller size has a higher particle retention ability [38,57], while a higher total leaf area of plants can accumulate more aerosol particles.

The specific leaf area was significantly affected by both plant species and habitats. *A. philoxeroides* in the terrestrial habitats, with the lowest specific leaf area, exhibited the highest particle retention. This is consistent with the finding that plants with a low specific leaf area can accumulate significantly more particles [58]. Leaves with a high specific leaf area tend to be large and thin, resulting in a high degree of overlap and reduced effective area for particle deposition [59,60]. The complex canopy structure of plants with a low specific leaf area result in lower wind speeds nearby, increasing the deposition efficiency of aerosol particles [61,62] and reducing the resuspension of deposited particles back into the atmosphere [58,59]. Additionally, leaves with a low width-to-length ratio are highly efficient at particle retention [57] and can sway less, reducing particle resuspension [23]. Furthermore, a narrow leaf shape can reduce convective dissipation, decrease the temperature difference between the leaf surface and the environment, and make particle attachment more stable [5].

The epicuticular wax of plant leaves is reported as one of the crucial factors influencing the retention of aerosol particles [63]. Xu et al. [64] investigated the characteristics of particles deposited on the leaves of 17 urban plants, and found that approximately 35% of the particles were deposited in the waxy layer of the plants. However, the results of this study indicate a negative correlation between the particle accumulation and wax content. For example, *H. vulgaris* in the aquatic habitats exhibited a higher wax content but deposited fewer fine particles than those in the terrestrial habitats, contradicting the findings of Przybysz et al. [65]. Conversely, *A. philoxeroides* in the aquatic habitats had a significantly higher wax content and retained more fine particles than those in the terrestrial habitats in this study (Figure 5), consistent with [13], who found that a high wax content contributes to particle retention, particularly for fine particles. However, the deposited particles on the leaf surface can degrade the wax or alter its structure and composition, which could explain the lower wax content of the plants in the terrestrial habitats [28,66,67]. Consequently, the plant leaves in the terrestrial habitats deposited more particles and had a lower wax content than those in the aquatic habitats.

Moreover, stomata contribute to the deposition of aerosol particles. Plants can regulate the stomatal density, stomatal area, and the dynamics of stomatal opening and closure [68,69]. Plants growing in aquatic habitats generally exhibit a higher stomatal density and larger stomatal area than those in terrestrial habitats. However, in this study, *A. philoxeroides* and *H. vulgaris* in the aquatic habitats showed a lower stomatal density and smaller stomatal area than those in the terrestrial habitats (Figure 7), aligning with the findings of Tao et al. [70] and Wang et al. [71]. Notably, the stomatal conductance of the plants in the aquatic habitats was higher than in the terrestrial habitats (Figure 6g), possibly because stomatal conductance is largely influenced by the degree of stomatal opening and closure [69]. Another explanation may be that plant leaves in terrestrial habitats accumulate a large amount of aerosol particles that clog the stomata, resulting in a low stomatal conductance. The results showed that the stomatal area was positively correlated with particulate accumulation in both the aquatic and terrestrial habitats (Figure 8). Prigioniero et al. [11] also found that the stomatal area positively affected the PM_10_ concentration uptaken by plants. Furthermore, this study demonstrated a positive association between the stomatal density and particulate accumulation in the terrestrial habitats, corroborating the previous findings by Weerakkody et al. [38] and Marien et al. [58]. However, these findings contradict the results of Chen et al. [13], possibly due to seasonal or leaf age effects, with younger leaves being more susceptible to the adverse impacts of particulate matter, leading to impaired stomatal development.

### 4.3. The Effects of Particle Deposition on the Physiological Features of Plants

Particle deposition exhibited notable stress on the physiological features of plants, especially their photosynthetic rate and transpiration rate, in both the terrestrial and aquatic habitats (Figure 6). Previous studies have demonstrated that particle accumulation adversely impacts plant photosynthesis [66,72]. For example, deposited particles affect the optical characteristics of plant leaves, decreasing photon absorption and utilization, which results in a decreased photosynthetic rate [28]. Additionally, fine particles and coarse particles can easily block or enter the stomata, which play a pivotal role in the gas exchange of plants [73]. This inhibits stomatal conductance and the transpiration rate of plant leaves, thereby reducing their photosynthetic rate [72]. Similarly, in this study, the transpiration and photosynthetic rates of both *A. philoxeroides* and *H. vulgaris* in the terrestrial or aquatic habitats were negatively correlated with the mass concentration of deposited particles. Increased particle deposition was closely associated with decreased transpiration and photosynthetic rates. Notably, Kong et al. [66] indicated that plant leaves with decreased transpiration rates facilitate the deposition of aerosol particles on leaf surfaces. This suggests that the reduction in transpiration rates caused by particle deposition further facilitates particle accumulation, thereby enhancing the stress on the plants.

This study not only provides insights into the negative effects of particle deposition on the physiological features of plant leaves, but also highlights the adaption strategies of plants against the stress of particle deposition. For example, there was a positive correlation between the mass concentration of deposited particles and the chlorophyll content of plant leaves, consistent with the findings of Popek et al. [72]. The reason may be that particle retention hinders plant photosynthesis, stimulating plant leaves to increase the chlorophyll content to maximize light absorption under PM-induced shading effects [74,75]. However, particle retention was negatively corrected with stomatal conductance. For instance, *A. philoxeroides* in the terrestrial habitats exhibited the highest particle retention and the lowest stomatal conductance, whereas *H. vulgaris* in the aquatic habitats had the lowest particle retention and the highest stomatal conductance. This indicates that particle deposition negatively affects plant growth, while plants adapt to this stress by reducing the stomatal conductance. Additionally, reducing the stomatal conductance can effectively prevent aerosol particles from entering the leaf tissue, which helps resist particle stress.

## 5. Conclusions

This study reveals that habitats significantly affected the size distribution and composition characteristics of aerosol particles deposited on the surfaces of plant leaves. Plants growing in terrestrial habitats deposited more large and coarse particles, most of which were primary particles rich in inorganic Ca^2+^, accounting for over 70% of total ions, mainly derived from the soil and ground dust. In contrast, the high relative humidity in aquatic habitats facilitated the deliquescence and deposition of fine particles and the generation of secondary particles, which had high fractions of water-soluble inorganic ions, especially NO_3_^−^ and SO_4_^2−^, taking up over 65% of total anions, possibly transformed from gaseous pollutants in the atmosphere. However, the mechanism behind the heterogeneous reactions of deposited particles in aquatic habitats, resulting in a low particle mass with high water-soluble inorganic ions, deserves further investigation. Furthermore, a more in-depth investigation is needed to characterize minor and trace elements in particulate matter and to understand their effects on plants.

The interactions between aerosol particles and plants exhibit some similarities in both terrestrial and aquatic habitats. Particle deposition negatively correlates with the leaf traits, including the leaf area, specific leaf area, and wax content. This indicates that plant leaves with a smaller leaf area, specific leaf area, and lower wax content tend to have a higher efficiency in retaining aerosol particles. Moreover, particle accumulation negatively affected the physiological features of the plants by inhibiting the stomatal conductance, reducing the transpiration rate, and decreasing the photosynthetic rate, which potentially facilitates further particle deposition and enhances stress. In brief, this study intensively assessed the differences in the characteristics of deposited particles and their effects on two plant species inhabiting both terrestrial and aquatic habitats, providing a reference for the scientific planning and configuration of urban greenery. To gain a comprehensive understanding of the role of aquatic plants in removing aerosol particles, further investigations should include additional plant species.

## Figures and Tables

**Table 1:** Two-way ANOVA on the composition characteristics of aerosol particles retained on *Alternanthera philoxeroides* and *Hydrocotyle vulgaris* in terrestrial and aquatic habitats.

Particle Characteristics	*F*_h_	*F_s_*	*F*_h_ × *F_s_*
Number density			
Fine particles	15.782 **	14.543 **	1.841
Coarse particles	0.012	19.381 ***	11.478 **
Large particles	0.781	22.464 ***	6.976 *
TSP	18.748 ***	58.196 ***	23.694 ***
Mass concentration			
Fine particles	12.952 **	7.942 **	12.648 **
Coarse particles	2.211	23.173 ***	1.592
Large particles	0.139	28.061 ***	12.432 **
TSP	13.823 **	64.499 ***	28.196 ***
Ion concentration			
Ca^2+^	0.029	0.555	0.848
K^+^	1.445	1.445	1.445
NH_4_^+^	1.497	1.896	2.834
Na^+^	1.079	1.201	1.079
SO_4_^2−^	8.497 *	1.178	2.304
NO_3_^−^	4.783 *	0.607	0.502
Cl^−^	0.036	3.363	0.036
F^−^	2.637	0.010	16.566 **

Notes: *F*_h_—treatment of different habitats (i.e., terrestrial and aquatic habitats); *F*_s_—treatment of different species (i.e., *A. philoxeroides* and *H. vulgaris*). *—*p* < 0.05; **—*p* < 0.01; ***—*p* < 0.001.

**Table 2:** Two-way ANOVA on the leaf functional traits of *A. philoxeroides* and *H. vulgaris* in terrestrial and aquatic habitats.

Leaf Functional Traits	*F*_h_	*F_s_*	*F*_h_ × *F_s_*
Leaf area	2.555	293.908 ***	0.038
Specific leaf area	48.463 ***	132.115 ***	4.714 *
Leaf biomass	1.996	117.595 ***	2.682
Leaf temperature	1.972	0.000	6.724 *
Wax content	23.660 ***	21.692 ***	2.376
Chlorophyll content	237.371 ***	652.221 ***	28.381 ***
Stomatal conductance	23.125 ***	296.199 ***	3.139
Stomatal density	211.170 ***	92.857 ***	143.989 ***
Stomatal area	833.937 ***	2987.058 ***	19.031 ***
Transpiration rate	35.022 ***	573.681 ***	0.134
Photosynthetic rate	248.322 ***	204.632 ***	6.168 *

Notes: *F*_h_—treatment of different habitats (i.e., terrestrial and aquatic habitats); *F*_s_—treatment of different species (i.e., *A. philoxeroides* and *H. vulgaris*). *—*p* < 0.05; ***—*p* < 0.001.

**Table 3:** Summary of the differences in particle characteristics and plant leaf traits between terrestrial and aquatic habitats.

Subjects	Parameters	Terrestrial Habitat	Aquatic Habitat
Particle characteristics	Particle size	More coarse and large particles	More fine particles, varied with the species
	Number density	A little higher, in the adaxial surface	A little lower, on the whole
	Mass concentration	Higher, in the adaxial surface	Lower, on the whole
	Micro-morphological characteristics	Larger, irregular	Smaller, regular
	Main types	Dominated by mineral particles	Most are secondary particles
	Water-soluble inorganic ions	Lower	Higher
Plant leaf traits	Growth traits	Normal	Better
	Morphological traits	Normal	Better
	Stomatal density	Higher	Lower
	Stomatal area	Larger	Smaller
	Stomatal conductance	Lower	Higher
	Chlorophyll content	Lower	Higher
	Transpiration rate	Lower	Higher
	Photosynthetic rate	Lower	Higher

## Data Availability

All of the data are presented in this paper.
